# Evaluating the Appropriateness of MRSA Nasal PCR Screening for De-escalation of MRSA-directed Therapy

**DOI:** 10.1017/ash.2025.275

**Published:** 2025-09-24

**Authors:** Warren Acker, Laken Smothers, Majd Alsoubani, Maureen Campion, Gabriela Andujar-Vazquez

**Affiliations:** 1 Tufts Medical Center; 2 Tufts University School of Medicine; 3 Tufts Medical Center; 4 Tufts Medical Center; 5 Dartmouth Hitchcock Medical Center

## Abstract

**Background:** Methicillin-resistant Staphylococcus aureus (MRSA) nasal polymerase chain reaction(PCR) is a rapid screening test (turnaround time ~1-2 hours) used to evaluate nasal colonization with MRSA. For respiratory and bloodstream infections, MRSA nasal swab has a high negative predictive value >95% which could help de-escalation of MRSA directed therapy in the appropriate clinical setting. Tufts Medical Center recently implemented this test and requires an indication for ordering: pneumonia, sepsis or septic shock, bacteremia, or other (with free-text reason). **Intervention/Aim:** Evaluate clinician adherence to the recommended indications through reviewing of ordering indication and assess utilization of MRSA PCR as a tool for de-escalating MRSA directed antibiotics. **Methods:** Retrospective review of MRSA PCR orders between September 28, 2023, and March 28, 2024. Ordering data including indication selected and test result was extracted from the electronic medical records. Other variables were collected by chart review by two study members. Free-text reasons documented when selecting “other” were categorized by system (e.g genitouniary or skin and soft tissue). Free text reasons were evaluated based upon published negative predictive value (NPV). Indications with NPV lower than 95% were considered inappropriate. MRSA antibiotics were considered vancomycin, daptomycin, linezolid, or ceftaroline. Changes in MRSA antibiotics were determined by chart review of the antibiotics administered at least 24 hours prior to MRSA PCR administration and 24 hours after administration. **Results:** 113 of 1339 tests were ordered with “other” indication. Only 17 (15%) of these orders were considered appropriate. Among the appropriate tests were infections involving the head, eyes, ears, nose, and throat (HEENT). Of 441 tests reviewed, 411 were negative (93.2%). Of those with negative tests 324 (78.8%) were given MRSA antibiotics prior to the test. and only 92 (28.4%) remained on MRSA therapy after a negative test **Conclusion:** Reviewing “others” helped identify gaps in knowledge to target educational interventions and identify additional appropriate indications to include in the computerized order entryMRSA Nares is an effective tool to de-escalate MRSA antibiotics, but other interventions are needed to increase appropriate antibiotic de-escalation with a negative test.

